# Adverse drug events associated with sodium zirconium cyclosilicate: A real-world pharmacovigilance study based on the FAERS database

**DOI:** 10.1371/journal.pone.0330340

**Published:** 2025-09-02

**Authors:** Yinrui Fang, Zilun Wu, Qian Wu, Gangyi Chen

**Affiliations:** 1 Guangzhou University of Chinese Medicine, Guangzhou, China; 2 The First Affiliated Hospital of Guangzhou University of Chinese Medicine, Guangzhou, China; 3 Guangdong Clinical Research Academy of Chinese Medicine, Guangzhou, China; Stellenbosch University Faculty of Medicine and Health Sciences, SOUTH AFRICA

## Abstract

**Background:**

Sodium zirconium cyclosilicate (SZC, Lokelma) is a novel hyperkalemia therapy, but comprehensive real-world safety data are lacking. This study aimed to characterize SZC-associated adverse events (AEs) using post-marketing surveillance.

**Research Design and Methods:**

AE reports for SZC/Lokelma were extracted from the FDA Adverse Event Reporting System (FAERS) (2004–2023). Four disproportionality algorithms (ROR, PRR, BCPNN, MGPS) identified safety signals. Significant system organ class (SOC) signals required ROR ≥ 2; preferred term (PT) signals met all algorithm thresholds, with false discovery rate adjustment.

**Results:**

Among 1,564 AE reports (49% males, 29.5% females), four SOCs showed significant signals: metabolism/nutrition, renal/urinary, cardiac, and general disorders. Eighteen PT signals included hypokalemia, cardiac failure, and hypertension. Previously unreported AEs (e.g., ileus, ventricular fibrillation) emerged. AEs peaked early (41.87% within 30 days). Subgroup analyses confirmed robustness.

**Conclusions:**

This study highlights both previously recognized and potentially novel adverse event signals associated with SZC, particularly during the early phase of treatment. While limited by the inherent constraints of spontaneous reporting systems—such as underreporting and missing data—our findings suggest that clinicians may consider closer monitoring of metabolic, renal, and cardiac adverse events during initial therapy. Observed early signals merit further validation in prospective studies, while long-term risks remain to be clarified.

## Introduction

The sodium zirconium cyclosilicate (SZC, brand name: Lokelma) is a relatively new medication that was approved by the US Food and Drug administration (FDA) in May 2018 for the treatment of hyperkalemia [1]. Through its ability to bind potassium ions in the gastrointestinal tract rather than being systemically absorbed, it can reduce hyperkalemia [2] while lowering the risks associated with systemic adverse effects. Multiple clinical trials, particularly phase III studies, have demonstrated the superior efficacy of SZC in the management of hyperkalemia. Trial results have shown that SZC can significantly lower serum potassium levels, and patients typically experience substantial improvements within 24 hours [3]. While numerous clinical studies have proven its efficacy in managing hyperkalemia, its real-world safety profile remains inadequately explored.

On one hand, the strict inclusion criteria and limited sample sizes of clinical trials often fail to fully capture the broader range of adverse events (AEs) that may occur in the general patient population. Moreover, while the product label may mention certain common AEs, rare or unexpected events may be overlooked, and the factors influencing the incidence and severity of these AEs (such as patient demographics, comorbidities, and concomitant medication use) warrant further investigation. Therefore, post-marketing surveillance is necessary to better identify the potential risks associated with the medication.

The FDA adverse event reporting system (FAERS), is a spontaneous reporting system developed by the US FDA to collect and store reports of adverse drug reactions (ADRs) [4], medication errors, and product quality complaints from healthcare professionals, consumers, manufacturers, and patients. This system is a crucial tool for pharmacovigilance, as the extensive data collection from multiple sources helps identify safety signals, assess drug risks, and manage AEs [5]. By analyzing this database, potential safety concerns with medications can be detected early, informing clinical management and risk mitigation strategies.

The primary objective of this study is to address the research gap in the safety profile of SZC by utilizing the FAERS database to identify and characterize the AEs associated with its use. The innovation of this study lies in the application of advanced signal detection algorithms, including the reporting odds ratio (ROR) [6], proportional reporting ratio (PRR) [7], bayesian confidence propagation neural network (BCPNN), and multi-item gamma poisson shrinker (MGPS) [8], to rigorously detect and evaluate the association strength between SZC and its AEs. This multi-algorithm approach ensures the accuracy and comprehensiveness of the drug safety assessment. Moreover, by exploring the safety profiles across different patient subgroups (such as gender and age), this study contributes new perspectives to the literature and provides novel insights into the real-world safety of SZC.

## Materials and methods

### Data source

AE reports from Q1 2004 to Q3 2024 were retrieved from the FAERS database using the keywords “Sodium zirconium cyclosilicate” or “Lokelma”. To provide a comprehensive comparative safety profile, we further conducted a disproportionality analysis comparing SZC with patiromer, another widely used potassium binder for hyperkalemia management. Using the FAERS database, we retrieved patiromer-associated AE reports for the same timeframe and applied identical statistical methods as described for SZC.

### Data processing

After the raw data was retrieved from FAERS, a series of preprocessing steps were implemented. The inclusion criteria for this study were as follows: (1) The study period spanned from 2004 to 2023, with data sourced from the FAERS database; (2) AE reports associated with the use of SZC were collected, encompassing both monotherapy and combination therapy reports. The exclusion criteria were: (1) Reports with missing, unclear, or inaccurate basic information; (2) Reports containing data errors or inconsistencies; (3) Duplicate reports, where reports with identical values in fields such as gender, age, country, event date, and adverse reactions were considered duplicates.

This study considered only reports in FAERS where SZC was identified as the “primary suspect (PS)” drug. AEs were encoded and classified using the preferred terms (PT) and system organ class (SOC) from the MedDRA 25.1 dictionary, in order to standardize event descriptions and facilitate subsequent statistical analysis.

### Statistical analysis

The study employed rigorous methods for evaluating ADRs. The proportional imbalance method and bayesian method are two commonly used tools in the field of pharmacovigilance. The proportional imbalance method assesses the safety of drugs by comparing the proportion of reported AEs between a specific drug and other drugs, focusing on the PRR and the ROR indicators [9,10]. In contrast, the bayesian method utilizes two core algorithms: BCPNN and the MGPS. The formulas for the algorithms and their signal detection criteria are detailed in Table 1. SOC signals with ROR ≥ 2 were considered significant. To improve the reliability of the analysis of the relationship between the drug and PTs, only positive signals that simultaneously meet the threshold criteria of the four algorithms were considered statistically significant [11]. For multiple positive signals associated with PTs, we applied the false discovery rate (FDR) approach to adjust for multiple comparisons. Statistical significance was defined as a two-tailed p-value less than 0.05.

**Table 1:** Formulas and criteria for signal detection algorithms in pharmacovigilance.

Algorithms	Equation	Criteria
ROR	ROR = ad / bc 95%CI = e^(ln (ROR) ±1.96(1 / a + 1 / b + 1 / c + 1 / d)^0.5)	ROR ≥ 2, N ≥ 3
PRR	PRR = a(c + d) / c / (a + b) χ² = [(ad – bc)^2](a + b + c + d) / [(a + b)(c + d)(a + c)(b + d)]	PRR ≥ 2, χ² ≥ 4, N ≥ 3
BCPNN	IC = log₂ a(a + b + c + d)(a + c)(a + b) 95%CI = E(IC) ± 2V(IC)^0.5	IC025 > 0
MGPS	EBGM = a(a + b + c + d) / (a + c) / (a + b) 95%CI = e^(ln (EBGM) ± 1.96(1 / a + 1 / b + 1 / c + 1 / d)^0.5)	EBGM05 > 2

ROR: reporting odds ratio; PRR: proportional reporting ratio; BCPNN: bayesian confidence propagation neural network; MGPS: multi-Item gamma poisson shrinker; a: the number of reports that contain both the target drug and the target ADR; b: the number of reports of the target drug and other adverse reactions; c: the number of reports containing the target adverse reaction and other drugs; d: the number of reports containing other drugs and adverse reactions; CI: confidence interval; N: the total number of reports; χ²: the chi-square test; IC: the information component, used to assess the strength of the association between drugs and adverse reactions; IC025: the lower limit of IC. If IC025 > 0, it indicates a significant positive association; E(IC): the expected value of IC; V(IC): the variance of IC; EBGM: the empirical Bayes geometric mean, used to stably estimate the strength of association.

**Fig 1:**
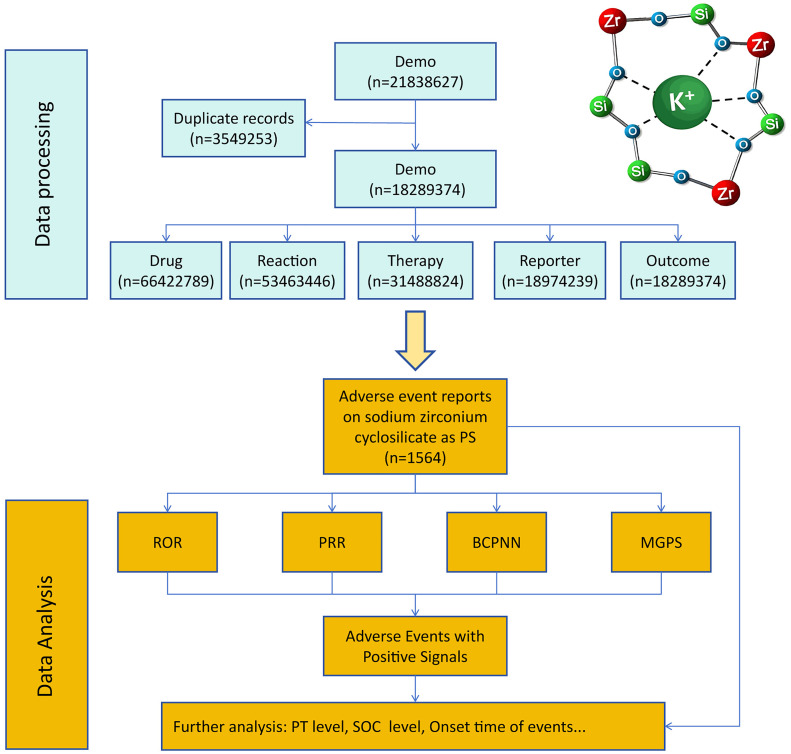
Flowchart of the research design in this study. ROR: reporting odds ratio; PRR: proportional reporting ratio; BCPNN: bayesian confidence propagation neural network; MGPS: multi-Item gamma poisson shrinker; PT: preferred term; SOC: system organ class.

The onset time was defined as the time interval between the START_DT (the date the drug was first used) and the EVENT_DT (the date the adverse event occurred). Additionally, subgroup analyses were performed to explore potential differences in the AE profiles across various patient groups, such as those stratified by age, gender, and comorbidities. All data analysis and visualizations for this study were conducted using R software (https://www.r-project.org/ ; version 4.3.4). The specific research design is presented in Fig 1.

### Ethics approval

The institutional review board of the First Affiliated Hospital of Guangzhou University of Chinese Medicine determined that approval was not required for this study, as it utilized publicly available data.

## Results

### Baseline characteristics

A total of 1,564 AEs associated with SZC were reported in the FAERS database (Table 2). Among the reports, 49% (767 cases) were male, 29.5% (462 cases) were female, and 21.4% (335 cases) had missing gender data. Age data were absent in 65.9% (1,031 reports), with 8.1% (126 reports) of the cases being under 65 years of age, 14.1% (221 reports) between the ages of 65 and 85 years, and 13.6% (212 reports) over the age of 85 years. The majority of reports were submitted by consumers (34.4%, 538 reports) and physicians (31.6%, 494 reports), with fewer reports submitted by pharmacists (13.8%, 216 reports) and other healthcare professionals (15.3%, 240 reports). Geographically, the highest proportion of reports originated from the United States (74.2%, 1,160 reports), followed by Japan (16.6%, 260 reports), and fewer reports came from Colombia, China, and the United Kingdom. The number of reports has shown a steady increase over the years, with the highest number of reports in 2023 (410 reports) and 2024 (417 reports). The complete data on baseline characteristics can be found in S1 Table.

**Table 2:** Baseline characteristics of the study.

Characteristic	Number	%
Overall	1564	100.00%
Sex		
Male	767	49.00%
Female	462	29.50%
Missing	335	21.40%
Age (years)		
< 65	126	
65 - 85	221	
> 85	212	
Missing	1031	65.90%
Reporting staff		
Consumer	538	34.40%
Physician	494	31.60%
Pharmacist	216	13.80%
Health Professional	240	15.30%
Others	5	0.30%
Missing	71	4.50%
Reporter country		
United States	1160	74.20%
Japan	260	16.60%
Colombia	34	2.20%
China	23	1.50%
United Kingdom	23	1.50%
Other countries	64	4.10%
Reported year		
2018	7	0.45%
2019	101	6.46%
2020	158	10.10%
2021	204	13.04%
2022	267	17.07%
2023	410	26.21%
2024	417	26.66%

### Signal detects at SOC Level

**Fig 2:**
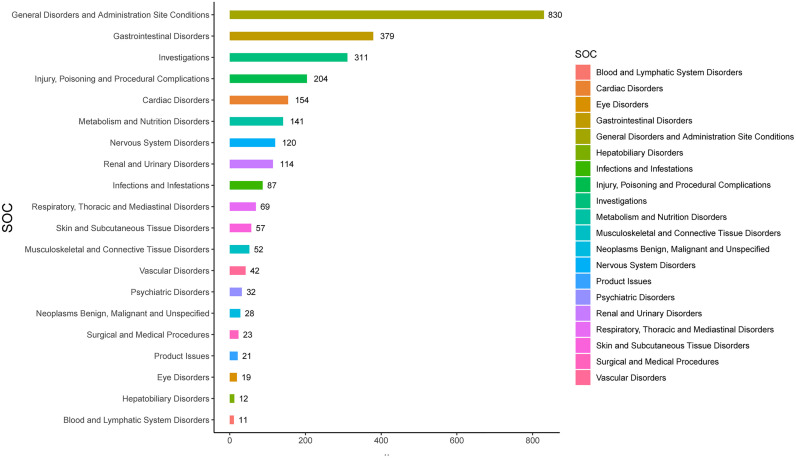
The number of adverse event reports at the SOC level. SOC: system organ class.

The AEs related to SZC are distributed across 26 SOCs, Fig 2 shows the top 20 SOCs arranged by quantity. In terms of the number of adverse event reports, general disorders and administration site conditions had 830 events, being one of the more frequently reported SOCs. Gastrointestinal disorders followed closely with 379 events. Injury, Poisoning and Procedural Complications and Cardiac Disorders were ranked third and fourth, respectively.

**Fig 3:**
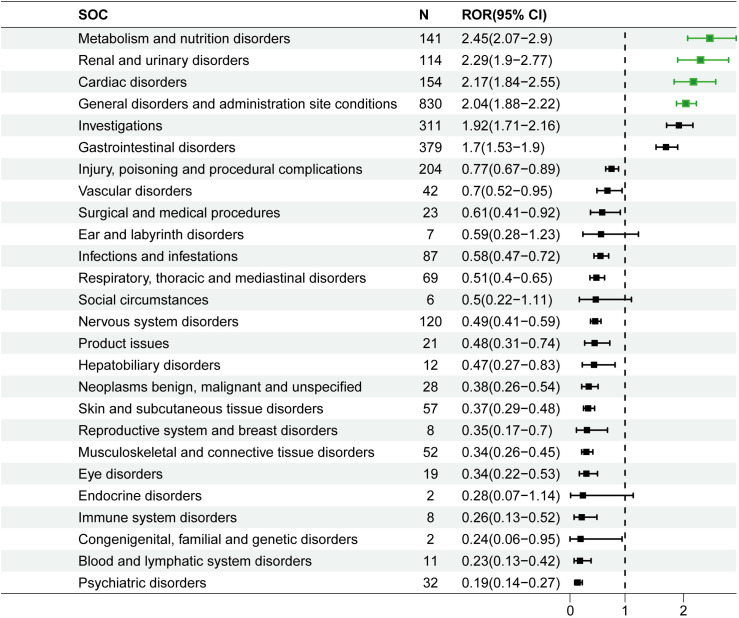
Forest plot of effect estimates for adverse-event reports at the SOC level. SOC: system organ class.

Regarding signal strength, it was found that there were 4 SOCs with an ROR ≥ 2 (Fig 3). Metabolism and nutrition disorders exhibited a relatively high ROR of 2.45 (95% confidence interval [CI]: 2.07–2.90), clearly indicating a significant positive signal. Renal and urinary disorders also showed a notable signal with an ROR of 2.29 (95% CI: 1.9–2.77). Cardiac disorders had an ROR of 2.17 (95% CI: 1.84–2.55), while gastrointestinal disorders had an ROR of 1.7 (95% CI: 1.53–1.9).

### Signal detects at PT level

**Fig 4:**
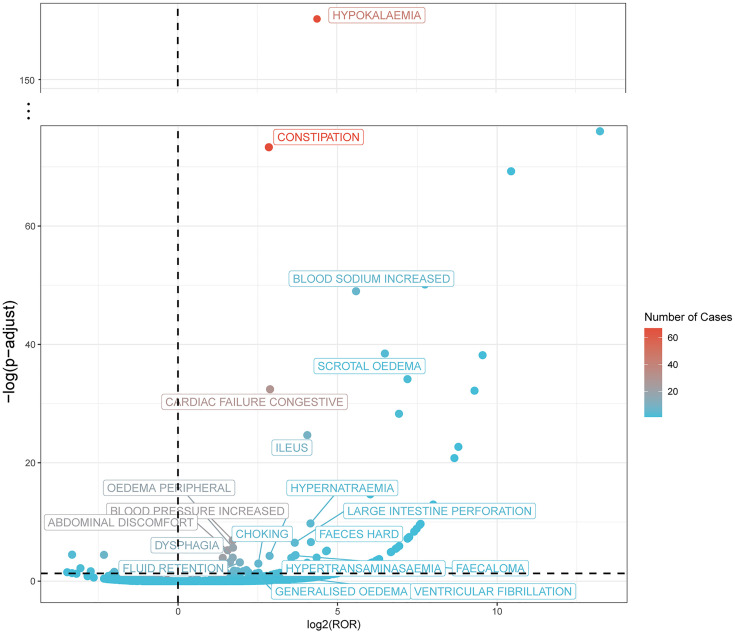
Volcano plot of PT signal distribution. Each dot represents a sodium zirconium cyclosilicate-related PT signal, and the color represents the number of reported PT signals. The results that are positive for all four algorithms are labeled.

In the signal detection of ADRs, 19 ADR signals were identified using four statistical methods (Fig 4; Table 3). After multiple test correction of the p-values from the chi-square test, the FDR for generalised oedema was 0.052, and thus it was excluded (S1 Table).

**Table 3:** The signal values of adverse reactions associated with sodium zirconium cyclosilicate based on preferred terms.

PT	Number	ROR (95%Cl)	PRR (X2)	EBGM (EBGM05)	IC (IC025)
Constipation	67	7.19 (5.64 - 9.16)	7.04 (348.03)	7.03 (5.74)	2.81 (2.46)
Hypokalaemia	43	20.99 (15.53 - 28.37)	20.67 (804.86)	20.65 (16.05)	4.37 (3.93)
Cardiac failure congestive	29	7.38 (5.12 - 10.65)	7.32 (158.33)	7.31 (5.39)	2.87 (2.34)
Blood pressure increased	23	3.28 (2.18 - 4.95)	3.26 (36.21)	3.26 (2.32)	1.71 (1.11)
Abdominal discomfort	22	2.94 (1.93 - 4.47)	2.92 (27.86)	2.92 (2.06)	1.55 (0.94)
Oedema peripheral	19	3.3 (2.1 - 5.19)	3.29 (30.28)	3.29 (2.25)	1.72 (1.07)
Dysphagia	14	3.28 (1.94 - 5.55)	3.27 (22.1)	3.27 (2.11)	1.71 (0.96)
Ileus	9	16.57 (8.61 - 31.9)	16.52 (131.16)	16.51 (9.55)	4.05 (3.13)
Fluid retention	9	3.83 (1.99 - 7.37)	3.82 (18.74)	3.82 (2.21)	1.93 (1.02)
Blood sodium increased	6	47.79 (21.43 - 106.57)	47.68 (273.57)	47.57 (24.32)	5.57 (4.48)
Choking	5	5.72 (2.38 - 13.76)	5.71 (19.44)	5.71 (2.74)	2.51 (1.33)
Large intestine perforation	4	12.62 (4.73 - 33.65)	12.6 (42.69)	12.59 (5.54)	3.65 (2.36)
Hypernatraemia	4	17.76 (6.66 - 47.38)	17.74 (63.13)	17.72 (7.8)	4.15 (2.86)
Scrotal oedema	3	89.47 (28.76 - 278.3)	89.37 (260.95)	88.97 (34.42)	6.48 (5.03)
Faeces hard	3	17.92 (5.77 - 55.63)	17.9 (47.83)	17.89 (6.93)	4.16 (2.72)
Faecaloma	3	12.63 (4.07 - 39.19)	12.61 (32.06)	12.61 (4.89)	3.66 (2.21)
Hypertransaminaemia	3	11.7 (3.77 - 36.31)	11.69 (29.3)	11.68 (4.53)	3.55 (2.1)
Ventricular fibrillation	3	5.77 (1.86 - 17.9)	5.76 (11.81)	5.76 (2.23)	2.53 (1.08)

PT, preferred terms; ROR, reporting odds ratio; PRR, proportional reporting ratio; χ2: chi – squared; IC: information component; IC025: the lower limit of 95% CI, of the IC; EBGM, empirical Bayesian geometric mean; EBGM05, the lower limit of 95% CI, of EBGM.

ADRs were observed across multiple body systems. In the gastrointestinal system, 67 cases of constipation were detected. The ROR value was 7.19 (95% confidence interval: 5.64–9.16), indicating a significant association between the use of SZC and the occurrence of constipation, suggesting a high statistical strength. Although there were only 9 cases of intestinal obstruction, the ROR value was as high as 16.57 (95% confidence interval: 8.61–31.9), suggesting a more significant association with drug use, which may have a severe impact on the gastrointestinal function of patients. Additionally, abdominal discomfort, hard stools, and fecal impaction were also detected as adverse reactions, indicating that the drug may interfere with the normal function of the gastrointestinal tract in multiple aspects.

Regarding electrolyte-related issues, 43 cases of hypokalemia were identified, with an ROR value of 20.99 (95% confidence interval: 15.53–28.37), suggesting a close relationship between drug use and the occurrence of hypokalemia. Signals of hypernatremia and increased blood sodium were also noteworthy. There were 4 cases of hypernatremia, with an ROR value of 17.76 (95% confidence interval: 6.66–47.38), and 6 cases of increased blood sodium, with an ROR value of 47.79 (95% confidence interval: 21.43–106.57). These data indicate that zirconium oxychloride may have a significant impact on the electrolyte balance of patients, and close monitoring of electrolyte levels is necessary during clinical use.

Regarding the cardiovascular system, 29 cases of congestive heart failure were found, with an ROR value of 7.38 (95% confidence interval: 5.12–10.65), suggesting that drug use may increase the risk of congestive heart failure. Additionally, signals of hypertension and ventricular fibrillation were detected. Although the number of cases was relatively small, this still indicates the potential impact of the drug on the cardiovascular system. Therefore, clinicians may need to remain vigilant for possible cardiovascular adverse events when using SZC, while awaiting further confirmatory studies.

Furthermore, positive signals were detected for peripheral edema, dysphagia, fluid retention, aspiration pneumonia, asphyxia, fractures, scrotal edema, and increased transaminases. These adverse reactions involve multiple body systems, indicating that the use of zirconium oxychloride in clinical practice may lead to a wide range of adverse reactions.

### Onset time of events

**Fig 5:**
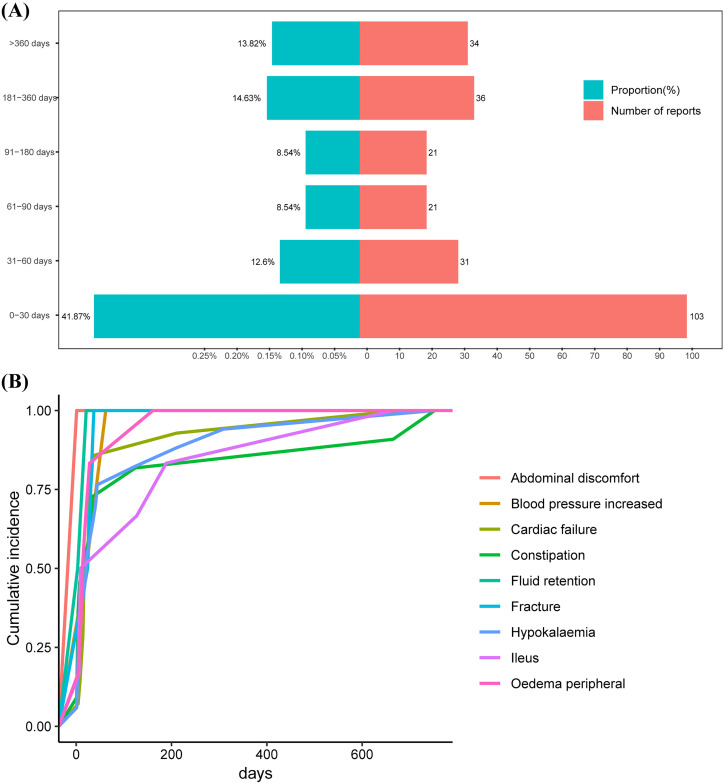
Onset time of adverse events associated with sodium zirconium cyclosilicate. **(A)** The onset time and the corresponding proportions of all adverse events. **(B)** Cumulative incidence curves of different adverse events.

Fig 5. (A) illustrates the distribution and corresponding proportions of AEs over different time periods. Within the 0-30 day interval, a total of 103 AEs were reported, accounting for a substantial proportion of 41.87%. In the 31-60 day period, 31 AEs were reported, comprising 12.8% of the total. In the 61-90 day and 91-180 day intervals, 21 AEs were recorded in each period, respectively, representing 8.54% each. As the time span extended to 181-360 days, 36 AEs were observed, accounting for 14.89%. Beyond 360 days, 34 AEs occurred, making up 13.82%. This indicates that AEs are not limited to the early stage of medication use but are widely distributed across a longer time frame, with a decreasing trend in the frequency of occurrence over time.

Fig 5 (B) presents the cumulative occurrence trends of the top 9 PTs of AEs. It is noteworthy that most AEs had achieved 100% of their cumulative occurrence within the first 50 days of medication use, such as abdominal discomfort, fluid retention, fracture, and blood pressure increase. This finding suggests that close monitoring for these AEs should be emphasized during the early stage of the medication regimen to enable timely detection and implementation of appropriate measures. In contrast, some AEs, such as Ileus and Constipation, may be delayed in their onset, occurring after 200 days. The complete onset time data can be seen in the S1 Table.

Notably, subgroup analysis of adverse events reported ≥365 days after SZC initiation (n = 34) revealed that none reached disproportionality thresholds (all EBGM05 < 2 and IC025 < 0, detailed data are provided in S1 Table), indicating no statistically significant late-onset safety signals in this cohort.

### Subgroup analysis

**Fig 6:**
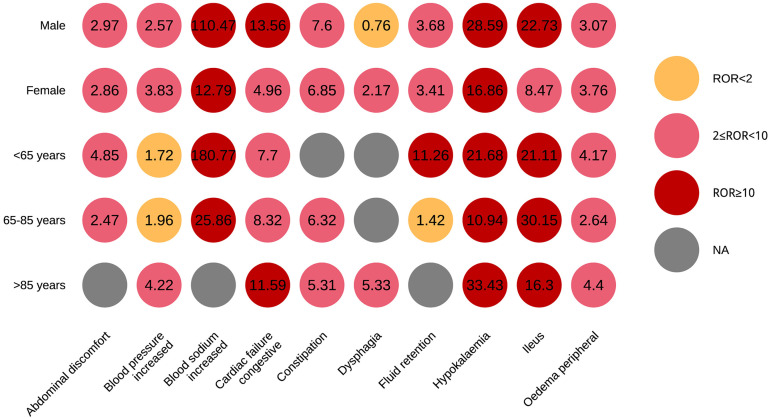
Subgroup analyses stratified by gender and age.

Subgroup analyses were conducted in this study to investigate the gender and age related differences in the top 10 AEs associated with SZC (Fig 6).

With regard to gender, males exhibited a significantly higher risk of hypernatremia when using SZC (ROR = 110.47, substantially higher than the ROR of 12.79 in females), warranting close monitoring of serum sodium levels. The ROR for hypokalemia was also higher in males (28.59) compared to females (16.86), suggesting that males are more prone to developing hypokalemia and require enhanced potassium monitoring. Furthermore, the ROR for heart failure was 13.56 in males, which was higher than the ROR of 4.96 in females, indicating that males may be at greater risk of cardiac impairment associated with SZC use, necessitating close attention to cardiac-related symptoms and parameters.

In terms of age differences, the < 65 years age subgroup displayed a remarkably elevated ROR of 180.77 for hypernatremia, substantially higher than other age groups, suggesting that younger patients experience a greater impact on sodium metabolism and should be closely monitored. The 65–85 years age group had a relatively high ROR of 30.15 for hypokalemia, underscoring the need to closely monitor serum potassium levels and associated symptoms in this age range. The > 85 years age subgroup exhibited the highest ROR of 33.43 for fluid retention, indicating an increased risk of edema and the need for close observation of fluid-related signs and timely adjustments to the treatment regimen in elderly patients.

Despite the observed differences in adverse events among gender and age subgroups, the majority of PTs for adverse events had ROR values of 2 or greater across all subgroups, demonstrating the robustness of the analysis. Targeted monitoring and management based on patient gender and age characteristics can effectively improve the clinical safety and efficacy of SZC use.

### Comparative disproportionality analysis between SZC and Patiromer

The disproportionality analysis identified 22 significant adverse-event PTs associated with patiromer (Table 4). Among these PTs, four were shared between patiromer and SZC: constipation, faeces hard, abdominal discomfort, and congestive heart failure. The remaining PTs were drug-specific, with SZC uniquely associated with events such as intestinal obstruction, hypernatremia, and ventricular fibrillation, and patiromer uniquely associated with events including diarrhea, hypo-/hyper-magnesemia, and hypercalcemia.

**Table 4:** The signal values of adverse reactions associated with patiromer based on preferred terms.

PT	Number	ROR (95%Cl)	PRR (X2)	EBGM (EBGM05)	IC (IC025)
Constipation	2 105	24.60 (23.53–25.73)	22.81 (43 533.55)	22.56 (21.73)	4.50 (4.43)
Diarrhoea	1 151	4.19 (3.95–4.45)	4.06 (2 674.56)	4.05 (3.86)	2.02 (1.93)
Blood potassium	763	112.09 (104.11–120.68)	109.03 (77 387.26)	103.34 (97.14)	6.69 (6.58)
Abdominal discomfort	442	5.87 (5.34–6.45)	5.79 (1 751.04)	5.78 (5.34)	2.53 (2.39)
Flatulence	362	14.75 (13.29–16.37)	14.57 (4 546.02)	14.47 (13.26)	3.86 (3.70)
Abdominal pain upper	322	3.58 (3.20–3.99)	3.55 (589.50)	3.54 (3.23)	1.82 (1.66)
Muscle spasms	243	2.90 (2.56–3.29)	2.89 (299.87)	2.88 (2.59)	1.53 (1.34)
Blood potassium	196	14.51 (12.60–16.71)	14.41 (2 430.02)	14.32 (12.72)	3.84 (3.63)
Abdominal distension	143	3.14 (2.67–3.70)	3.13 (207.38)	3.13 (2.73)	1.64 (1.40)
Cardiac failure congestive	122	3.11 (2.60–3.71)	3.10 (173.18)	3.09 (2.67)	1.63 (1.37)
Hyperkalaemia	75	4.81 (3.83–6.03)	4.80 (225.12)	4.79 (3.96)	2.26 (1.93)
Faeces hard	71	45.02 (35.57–56.98)	44.91 (2 979.97)	43.92 (36.07)	5.46 (5.11)
Blood magnesium	59	15.53 (12.02–20.07)	15.50 (794.21)	15.39 (12.42)	3.94 (3.57)
Retching	57	6.04 (4.66–7.84)	6.03 (238.52)	6.01 (4.84)	2.59 (2.21)
Blood calcium	34	8.67 (6.19–12.14)	8.66 (229.28)	8.62 (6.50)	3.11 (2.62)
Blood pressure abnormal	28	3.29 (2.27–4.77)	3.29 (44.62)	3.29 (2.41)	1.72 (1.18)
Hypercalcaemia	26	4.70 (3.20–6.90)	4.69 (75.39)	4.68 (3.39)	2.23 (1.67)
Blood phosphorus	17	14.63 (9.08–23.59)	14.63 (214.22)	14.53 (9.74)	3.86 (3.18)
Blood iron	16	3.26 (1.99–5.32)	3.26 (24.96)	3.25 (2.16)	1.70 (1.00)
Dyschezia	14	9.12 (5.40–15.43)	9.12 (100.75)	9.08 (5.85)	3.18 (2.44)
Faecal volume increased	4	17.83 (6.66–47.72)	17.83 (62.95)	17.67 (7.75)	4.14 (2.85)
Sickle cell anaemia	4	12.57 (4.70–33.60)	12.57 (42.32)	12.50 (5.49)	3.64 (2.35)

PT, preferred terms; ROR, reporting odds ratio; PRR, proportional reporting ratio; χ2: chi – squared; IC: information component; IC025: the lower limit of 95% CI, of the IC; EBGM, empirical Bayesian geometric mean; EBGM05, the lower limit of 95% CI, of EBGM.

**Fig 7:**
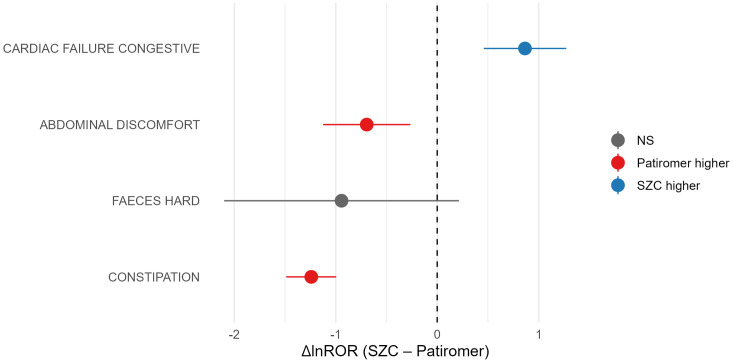
Comparison of disproportionality signals for shared adverse events between SZC and patiromer.

Quantitative analysis of the four shared PTs revealed significant differences in their relative association strengths between the two drugs (Fig 7). Congestive heart failure was more strongly associated with SZC (ΔlnROR = 0.87; 95% CI: 0.46–1.27, p < 0.001), while constipation (ΔlnROR = −1.24; 95% CI: −1.49–-0.99, p < 0.001) and abdominal discomfort (ΔlnROR = −0.70; 95% CI: −1.12–-0.27, p = 0.001) exhibited stronger associations with patiromer. No significant difference was observed for faeces hard (ΔlnROR = −0.94; 95% CI: −2.10–0.21, p = 0.16).

## Discussion

This study aims to comprehensively assess the safety profile of SZC, through the analysis of the FAERS database. Four algorithms—ROR, PRR, BCPNN, and MGPS—were employed to ensure the accuracy of the results, and subgroup analyses [12] were conducted to explore the differences in AEs based on various patient characteristics.

Previous research on SZC has predominantly focused on its efficacy in treating hyperkalemia [13], with limited attention given to its real-world safety. As a relatively new treatment for hyperkalemia, AE studies on SZC have largely relied on Phase I, II, and III clinical trials [14,15,16] and case reports. However, emerging drugs, stringent trial designs, and strict inclusion criteria may lead to underreporting of rare AEs, resulting in incomplete drug safety information. This study leverages the FAERS database [17] to supplement the safety data for SZC in the real-world context. Compared to clinical trials, this study includes a broader and more diverse patient population, overcoming the limitations imposed by the stringent inclusion criteria and small sample sizes typically seen in clinical trials. This approach allows for the identification of a wider range of potential AEs. For instance, rare but serious events such as bowel obstruction and arrhythmias, which may not have been adequately captured in clinical trials, were identified through the analysis of a large number of real-world reports in this study.

SZC, developed by AstraZeneca, received a recommendation for marketing authorization from the Committee for Medicinal Products for Human Use (CHMP) of the European Medicines Agency (EMA) on February 24, 2017, and was subsequently approved by the European Union on March 22, 2018, for the treatment of hyperkalemia. This timeline aligns with the earliest reported AEs associated with SZC. Due to the limited number of countries initially approving SZC and the delayed reporting of AEs, only seven AE reports were collected in 2018. Over the past seven years, particularly since 2019, the incidence of SZC-related AEs and the number of reported events have shown a steady increase. This trend can likely be attributed to several factors: 1 the expansion of SZC’s indications and its growing usage. Initially approved as a K+ selective ion trap for lowering serum potassium, SZC has since been extended to the treatment of heart failure, renal failure, metabolic acidosis, and other conditions [18]. 2) The gradual acceptance of SZC by national governments and the dissemination of updated clinical guidelines by expert physicians. For instance, in November 2021, the FDA granted SZC Fast Track Designation (FTD) for the treatment of recurrent hyperkalemia in patients undergoing chronic dialysis, aiming to reduce arrhythmia-related cardiovascular outcomes. Following this, in August 2024, U.S. nephrologists updated treatment guidelines to include SZC for renal failure [19]. These developments have significantly increased the drug’s clinical influence. 3) Improvements in the adverse event reporting and recording systems, leading to more comprehensive AE data being uploaded to the FAERS database.

At the SOC level, disorders related to metabolism and nutrition, renal and urinary systems, cardiac conditions, and general disorders and administration site conditions have been identified as associated with SZC. Among these, metabolism and nutrition disorders exhibited the highest signal strength, with a ROR of 2.45 (95% CI: 2.07–2.9). This is also one of the most frequently mentioned adverse reactions in the SZC product label, primarily related to electrolyte imbalances such as hypokalemia and hypernatremia. These effects are likely due to the drug’s regulation of potassium ions and the possible accompanying sodium exchange mechanisms. Specifically, SZC is an oral potassium binder that is poorly absorbed by the body, with nearly 99% of it excreted. It selectively binds potassium ions throughout the gastrointestinal tract and exchanges them for hydrogen ions and sodium ions. This process effectively reduces the concentration of free potassium in the gastrointestinal tract and increases fecal potassium excretion, ultimately lowering serum potassium levels, thus treating hyperkalemia [20]. However, because SZC has a high potassium binding capacity—approximately nine times that of organic polymer resins such as sodium polystyrene sulfonate—it has a higher potassium clearance rate, which increases the risk of inducing hypokalemia. This risk is particularly pronounced in patients with end-stage renal disease (ESRD) on dialysis. Compared to placebo, patients using SZC demonstrated a higher incidence of hypokalemia following dialysis. However, the consequences are typically not severe, as measured by the median and range of post-dialysis serum potassium concentrations, with similar severity between the SZC and placebo groups [21]. In patients undergoing maintenance hemodialysis, post-dialysis hypokalemia is usually transient, and SZC-related pre-dialysis hypokalemia is generally not severe. Nonetheless, it is important to consider that hypokalemia induced by concomitant medications cannot be ruled out, as most patients using SZC also take loop diuretics or thiazide diuretics for hypertension management. These medications could confound the occurrence of reported adverse events related to hypokalemia. Additionally, many of the adverse event reports lacked a standardized definition for the concentration range of hypokalemia, which further complicates the assessment of whether SZC induces hypokalemia.

Regarding renal and urinary system adverse events, the primary reports currently pertain to urinary tract infections and renal injury. A clinical trial conducted in Phase II and III, which evaluated the combined adverse events of Patiromer and SZC, indicated a urinary tract infection incidence of 1.9% [22]. However, the specific reasons for this were not explained. It should be noted that for patients with renal failure, the use of SZC in the renal and urinary systems may offer more benefits than risks. One such potential benefit is its modest effect on increasing serum bicarbonate, which could be particularly helpful in managing chronic metabolic acidosis in patients with CKD or those receiving maintenance hemodialysis.

According to Wesson DE et al. [23], this effect is likely mediated through a gut–liver mechanism rather than renal ammoniagenesis. Specifically, SZC binds free ammonium ions (NH₄⁺) in the intestinal lumen—where NH₄ ⁺ closely mimics the ionic radius of K ⁺ —and promotes their fecal excretion. This reduces the portal ammonia load, thereby lowering hepatic nitrogen burden. In turn, the liver increases urea synthesis, a metabolic process that consumes hydrogen ions (H⁺) and generates new bicarbonate. For patients with impaired renal function—who have diminished or absent renal ammoniagenesis—this extra-renal pathway may help partially restore acid–base balance and elevate serum bicarbonate levels [24], ultimately contributing to better electrolyte and metabolic control in selected clinical contexts.

In accordance with the SOC definition and research data, the “general disorders and administration site conditions” in this study primarily refer to edema, which holds particular clinical significance due to the sodium content in SZC. Previous reports have observed a higher incidence of edema with higher SZC doses. A clinical trial revealed that participants receiving SZC at doses greater than 15g had a higher incidence of edema [25] compared to those receiving a placebo. However, the association between higher SZC doses and edema may be confounded by underlying primary conditions of the patients: those requiring higher SZC doses are more likely to have multiple comorbidities that contribute to edema, including lower estimated glomerular filtration rate (eGFR) and elevated baseline brain natriuretic peptide (BNP) levels.

At the PT signal level, a total of 21 ADR signals associated with SZC were identified, spanning various systems including gastrointestinal, electrolyte, and cardiovascular. Among these, gastrointestinal adverse reactions such as constipation, diarrhea, nausea, and vomiting were the most commonly observed. A multicenter, two-phase, double-blind, phase 3 trial involving 753 hyperkalemia patients revealed that during the maintenance phase [26], 25.1% of SZC patients and 24.5% of placebo patients reported adverse events. Diarrhea was the most frequently reported adverse event across all dosage levels and both study phases. In the initial phase, the incidence rate was 1.8% for SZC and 2.5% for the placebo group, while in the maintenance phase, it was 1.7% for SZC and 2.2% for placebo. Gastroenteritis was identified as the only serious adverse event potentially related to the medication, as suggested by Packham DK et al. It should be noted that in our research results, 60 cases of diarrhea were reported, and the three algorithms, ROR, PRR, and BCPNN, were positive. However, as the EBGM05 was less than 2, it was determined to not meet the MGPS algorithm criteria, and out of respect for the rigor of the statistical results, it was excluded from the positive findings. Nonetheless, based on previous studies and the available data [27], it is still believed that diarrhea may be one of the most common adverse reactions associated with SZC.

Our study also identified PT signals related to abdominal discomfort, hard stools, fecal impaction, and intestinal obstruction in patients using SZC. SZC functions by binding potassium ions in the gastrointestinal tract, which may explain the frequent occurrence of gastrointestinal adverse events. On one hand, SZC is not absorbed in the gastrointestinal tract, and chronic irritation of the gastrointestinal mucosa may affect intestinal motility. On the other hand, the binding of potassium ions by the drug may influence electrolyte levels within the gastrointestinal system, disrupting normal physiological functions and potentially leading to constipation or even intestinal obstruction.

Cardiovascular adverse reactions are another critical area requiring attention. On one hand, the use of SZC may lead to adverse events associated with edema, which increases peripheral volume load on the heart, potentially exacerbating conditions such as congestive heart failure, especially in the presence of electrolyte imbalances [28]. On the other hand, our study also observed potential PT signals of ventricular fibrillation, which may be related to electrophysiological changes induced by SZC, such as QT interval prolongation. The report mentioned above indicated that, in the initial phase, patients in the SZC group showed a corrected QT interval prolongation [26], consistent with decreased serum potassium levels, and this prolongation was dose-dependent. The average QT interval increase ranged from 0.03 milliseconds for the lowest SZC dose (1.25 g) on day 2 of the study, to 7.61 milliseconds for the highest SZC dose (10 g). However, the occurrence of clinically significant arrhythmias did not show a substantial difference between the SZC and placebo groups. In our analysis, the signal for congestive heart failure emerged as statistically significant across all four pharmacovigilance algorithms (ROR, PRR, IC, EBGM), even after false-discovery-rate correction. This association also appeared stronger for SZC than for patiromer. We propose that this finding, while consistent with prior reports of edema in pivotal trials, reflects a more severe clinical manifestation now apparent in real-world data. Specifically, the pivotal HARMONIZE trial reported a dose-related increase in edema, but patients with unstable NYHA class III–IV heart failure were largely excluded. In contrast, our analysis includes a broader, more vulnerable patient population. As such, similar fluid-retention effects may present as clinically coded exacerbations of CCF, which were not adjudicated as formal endpoints in the original trials. Therefore, the “novelty” of this signal lies in its emergence as a coded diagnosis—rather than an isolated symptom—in a real-world setting. Mechanistically, we consider two plausible contributors. First, the unavoidable sodium load: each 5 g dose of SZC contains ~400 mg of sodium, translating to 800–2,400 mg/day depending on treatment phase. For patients already advised to restrict sodium intake to <2 g/day, this may promote volume expansion, elevate cardiac preload, and precipitate heart failure decompensation. Second, potential drug–drug interactions: SZC, as a cation-exchange resin, may reduce gastrointestinal absorption of co-administered heart failure medications—particularly if dosing intervals are not appropriately spaced. While less frequent, this interference may further impair hemodynamic stability. Taken together, these mechanisms support cautious monitoring for volume-related complications, especially in elderly patients or those with baseline cardiovascular compromise.

Our analysis showed a median AE onset of 92 days, with 41.9% of events occurring within the first 30 days of SZC initiation. While 13.8% of AEs were reported after one year, subgroup analysis of reports ≥365 days post-initiation (n = 34) revealed no disproportionality signals (all EBGM05 < 2 and IC025 < 0). Cumulative incidence curves indicated that early events (≤60 days) predominantly included abdominal discomfort, elevated blood pressure, fluid retention, and fractures, whereas later-onset events (after 200 days) comprised cardiac failure, constipation, hypokalaemia, and ileus. Although causality cannot be firmly established from spontaneous reports, these data support vigilant monitoring for early-onset AEs and ongoing awareness of potential delayed reactions during long-term SZC therapy.

To provide a more comprehensive safety assessment of SZC, we conducted a head-to-head comparative safety analysis with patiromer, another leading second-generation potassium binder. Although both drugs are considered safe for treating hyperkalemia, their preferred clinical applications differ. Previous studies suggest that SZC is the preferred agent in acute hyperkalemia due to its more rapid onset of action, whereas patiromer is often favored for chronic management [29].

Despite both being non-absorbed polymers, their mechanisms of action are fundamentally different: SZC exchanges sodium and hydrogen ions for potassium, while patiromer exchanges calcium ions [29]. This mechanistic distinction implies distinct AE profiles related to electrolyte disturbances. Our findings confirm this, showing that patiromer was uniquely associated with hypo/hypermagnesemia and hypercalcemia, whereas SZC was associated with hypernatremia. These imbalances are mechanistically driven by the respective counter-ions exchanged for potassium.

Furthermore, SZC and patiromer exhibit different primary organ system toxicities. Our study confirmed that while both can affect the cardiac and gastrointestinal (GI) systems—sharing four PT signals for constipation, hard feces, abdominal discomfort, and congestive heart failure—their predilections vary. The primary AEs for SZC were cardiac-related. Its mechanism, involving sodium exchange, can increase the body’s sodium load, leading to edema and forming the basis for major cardiovascular safety concerns like congestive heart failure. Moreover, the rapid potassium-lowering effect of SZC can impact the cardiac QTc interval, potentially leading to unique events such as ventricular fibrillation, a finding consistent with our results. In contrast, patiromer’s GI side effects were more pronounced. Our analysis revealed significantly stronger signals for constipation (ΔlnROR = −1.24) and abdominal discomfort (ΔlnROR = −0.70) compared to SZC. This aligns with the higher incidence rates reported in clinical trials (e.g., constipation, 7.6%) [30]. Its potential for non-selective binding, leading to hypomagnesemia (5.3% incidence), also necessitates routine magnesium monitoring [31].

However, our study identified a unique and more severe AE signal for SZC: intestinal obstruction. Although this signal originated from a spontaneous reporting system, recent literature lends it credence. Case reports have described sigmoid colon perforation in patients treated with SZC, particularly those with underlying impaired intestinal transit [32]. Additionally, a large retrospective study in hospitalized veterans [33] found that SZC use was significantly associated with a risk of intestinal ischemia/thrombosis (adjusted odds ratio [aOR] = 1.78), despite a low overall incidence. In our study, patiromer did not show a statistically significant risk for such events.

It is noteworthy that AEs associated with SZC are more prevalent in males, with an incidence 1.67 times higher than in females, and the elderly population is more commonly affected. This may be attributable to the epidemiological characteristics of the conditions that SZC is used to treat. Epidemiological studies have shown that males are generally more exposed to certain risk factors, such as smoking and alcohol consumption, compared to females. As a result, the risk factors for conditions like renal failure, hypertension, and heart failure are significantly higher in males than in females [34,35]. Similarly, with regard to age, older individuals are more susceptible to hyperkalemia and cardiovascular and renal diseases, which explains why AEs are more frequently observed in patients over 65 years of age compared to younger adults. Furthermore, the majority of AE reports have come from the United States, followed by Japan. This distribution is less related to epidemiological factors and more to differences in the policies surrounding SZC use, the extent of clinical adoption in different countries, and the completeness of adverse event reporting systems. It is anticipated that as SZC becomes more widely used, particularly in developing countries, more AE data will be reported, ultimately contributing to a more comprehensive global real-world outcome prediction.

This study has certain limitations. First, the FAERS database is a spontaneous reporting system, which may introduce reporting biases that could lead to an underestimation or overestimation of the incidence of adverse events associated with SZC. For instance, mild adverse events may go unreported, while non-severe events may be over-reported [36]. Secondly, the database may contain incomplete or inaccurate information. Despite rigorous data preprocessing in this study, it remains challenging to entirely eliminate such issues. Furthermore, due to the nature of the data, while the analysis revealed a statistical correlation between the drug and adverse events, it does not establish causality [27]. The relationship between the drug and adverse events remains observational, and causal inference requires further validation. Moreover, since SZC is a relatively new drug, the sample size of adverse event reports is limited, and the results obtained may not fully reflect real-world outcomes. Potential adverse effects of SZC may still need time to be identified. Finally, this study is based solely on the FAERS database from the United States, with the majority of adverse event reports originating from the United States and Japan. Therefore, the findings may not be entirely generalizable to other countries or regions.

## Conclusion

The FAERS database was comprehensively analyzed to investigate the adverse events AEs associated with SZC. The analysis identified common AEs consistent with existing knowledge, including gastrointestinal disorders such as constipation. Additionally, novel AEs, such as hypernatremia and congestive heart failure, were discovered. These findings provide supplementary information regarding the relevant adverse reactions in the product labeling and offer objective evidence for the safe clinical use of the medication. This study emphasizes the significance of continuous monitoring and investigation of SZC-related AEs to better understand its safety profile, optimize clinical utilization, and improve patient care. In the future, large-scale, rigorous epidemiological studies should be relied upon to obtain more comprehensive evidence.

## Supporting information

S1 Table Summary of baseline characteristics, disproportionality analysis, onset-time distribution, and additional analyses for SZC adverse events in FAERS. (XLSX)
